# Comparison of Growth and Metabolomic Profiles of Two Afforestation Cypress Species *Cupressus chengiana* and *Platycladus orientalis* Grown at Minjiang Valley in Southwest China

**DOI:** 10.3390/metabo14080453

**Published:** 2024-08-17

**Authors:** Zhengqiao Liao, Lijun Zhu, Lei Liu, Jürgen Kreuzwieser, Christiane Werner, Baoguo Du

**Affiliations:** 1 College of Life Science and Biotechnology, Mianyang Normal University, Mianxing Road West 166, Mianyang 621000, China; zqliao@mtc.edu.cn (Z.L.); zhulijun211@mtc.edu.cn (L.Z.); liulei@mtc.edu.cn (L.L.); 2 Ecological Security and Protection Key Laboratory of Sichuan Province, Mianyang Normal University, Mianxing Road West 166, Mianyang 621000, China; 3 Chair of Ecosystem Physiology, Institute of Forest Sciences, University of Freiburg, Georges-Köhler-Allee 53, D-79110 Freiburg, Germany; juergen.kreuzwieser@ctp.uni-freiburg.de (J.K.); c.werner@cep.uni-freiburg.de (C.W.)

**Keywords:** dryland, carbohydrate, nitrogen, secondary metabolism, metabolome, soil, growth, afforestation, sugar, amino acid

## Abstract

In recent years, afforestation has been conducted in China’s hot and dry valleys. However, there is still a paucity of knowledge regarding the performance of tree species in these semi-arid regions, particularly with regard to interspecies differences. The present study compares the growth and metabolome characteristics of two widely used cypress species, namely *Cupressus chengiana* and *Platycladus orientalis*, grown at two sites with distinct climate conditions in the hot and dry Minjiang Valley in southwestern China. The findings indicate that *C. chengiana* trees exhibit superior growth rates compared to *P. orientalis* trees at both study sites. In comparison to *P. orientalis* trees, *C. chengiana* trees demonstrated a greater tendency to close their stomata in order to prevent water loss at the hotter and drier site, Llianghekou (LHK). Additionally, *C. chengiana* trees exhibited significantly lower hydrogen peroxide levels than *P. orientalis* trees, either due to lower production and/or higher scavenging of reactive oxygen species. *C. chengiana* trees accumulated soluble sugars as well as sugar derivatives, particularly those involved in sucrose and galactose metabolisms under stressful conditions. The species-specific differences were also reflected in metabolites involved in the tricarboxylic acid cycle, nitrogen, and secondary metabolisms. The metabolome profiles of the two species appeared to be influenced by the prevailing climatic conditions. It appeared that the trees at the drier and hotter site, LHK, were capable of efficient nitrogen uptake from the soil despite the low soil nitrogen concentration. This study is the first to compare the growth performance and metabolic profiles of two widely used tree species with high resistance to adverse conditions. In addition to the species-specific differences and adaptations to different sites, the present study also provides insights into potential management strategies to alleviate abiotic stress, particularly with regard to nitrogen nutrients, in the context of climate change.

## 1. Introduction

Drylands constitute about 45% of the world’s land surface [1,2]. Current projections indicate that this region will continue to expand by approximately 5.8 × 10^6^ km^2^ by the end of this century [3]. Forestation of the drylands has been considered a promising strategy to enhance carbon sequestration and benefits to the human environment. However, afforestation in these water-limited areas is a global challenge, particularly with increasing climate change [4]. In China, the large area of dry valleys along the upper streams of several rivers—for instance, the Yangtz, Lanchang, and Nu rivers, which surround the south and east Tibetan Plateau and are characterized by complex mountainous topography and typical hot and dry climate—is ecologically vulnerable and often affected by monsoon and foehn winds [5,6]. Since the 1990s, a series of ecological protection and restoration programs have been implemented by the Chinese government to improve the vegetation along the dry valleys. In the last decades, afforestation in these vulnerable areas has attracted a significant amount of local and international investment as an effective strategy for increasing carbon sequestration and mitigating the consequences of climate change [7,8,9,10].

The Minjiang River is a first-order branch of the Yangtze River; its upper-reach valley between the Tibetan Plateau and Sichuan Basin is a typical dry and hot belt with a length of 340 km and an area of about 23,000 km^2^ [11]. A drying trend with increasing numbers of heavy rainfall events is projected between 2030 and 2059 in the dry valley area [12]. Therefore, information on plants’ performance and resistance to projected dry and hot conditions is of particular importance for vegetation restoration and management. Previous studies were mainly focused on the responses of shrubs and grassland [13,14]; little is known about the tree species planted in this area in response to a dryer and hotter climate. The ability of tree species to cope with the dry and hot climate in these areas with shallow and poor soil is crucial to growth, development, and successful afforestation [8,9,10,15,16]. *Cupressus chengiana* and *Platycladus orientalis* are both long-lived, slow-growing evergreen tree species [17,18]. *C. chengiana* is listed as a “Second-Class Endangered Plant of China” and classified as “Vulnerable” by the IUCN. This species is naturally endemic to the northern Heng-duan mountain region [19]. *P. orientalis* is a widespread species native to China, and it is one of the most important afforesting tree species in northern China, especially in barren montane areas [18]. They have been widely planted in the last decades for afforestation in the dry valleys due to their high resistance to harsh environmental conditions such as drought, high temperature, and poor soil nutrients [17,18], and now have become the dominant tree species in the hot and dry Minjiang valley [20]. However, there is still little information on the differences in performance between the two species and their physiological responses to the hassle conditions.

In the present study, we investigate the growth pattern and their underlying biochemical mechanism of *Cupressus chengiana* and *Platycladus orientalis* from two sites with distinct water and temperature conditions. Specifically, we aimed to evaluate the growth performance, determine the metabolic similarity and difference of the two species in response to dry and hot climates, and provide information on afforestation in drylands in a projected warmer and drier climate.

## 2. Materials and Methods

### 2.1. Field Sites

The two field sites are located in the dry and hot valley (between 31°26′−33°16′ N and 102°59′−104°14′ E) of the upper Minjiang River, which is one of the four principal tributaries of the Yangtze River. The total area of the dry valley is about 23,000 km^2^ with a length of 340 km across Songpan, Heishui, Mao, Li, and Wenchuan counties on the eastern edge of Qinghai–Tibet Plain [11]. This region has a typical semi-arid continental monsoon climate. We selected two field sites with distinct climate conditions, i.e., the drier and hotter site Lianghekou (LHK, 31°50′ N,103°42′ E) located in the core area of the hot and dry Minjiang Valley, and one cool and wet site Cuojishan (CJS, 31°41′ N,103°51′ E) located in the edge of the hot and dry Minjiang valley. The elevation of LHK and CJS is 1820 m and 1655 m above sea level, respectively. Mean annual precipitation and air temperature were 369 mm and 13.3 °C at LHK and 574 mm and 10.6 °C at CJS, respectively [21]. The trees of *Cupressus chengiana* and *Platycladus orientalis* at CJS were planted in 2006, while they were planted in 2017 at LHK, both with 2-year-old seedlings of each species. Besides *C. chengiana* and *P. orientalis*, no other tree species were observed. The dominant shrub and grass species at LHK were *Sophora viciifolia*, *Bauhinia faberi var*. *microphylla,* and *Ajania fruticulose*. The dominant shrub and grass species at CJS were *Sophora viciifolia*, *Jasminum humile,* and *Ajania fruticulose*.

### 2.2. Tree Selection, Growth Measurement, and Leaf and Soil Sampling

Plant and soil sampling took place in October 2021. Leaf samples were harvested from six individual trees of each species at each site. For this purpose, a south-side twig at the middle crown was cut down, and leaves were immediately harvested and frozen in liquid nitrogen. The leaf samples were transferred to the lab under frozen and homogenized with mortar and pestle in liquid nitrogen and then stored at −80 °C until biochemical analysis. Soil samples were collected in two layers, 0–20 cm, and 20–40 cm, using a hand auger at the LHK and CJS sites. The litter was manually removed before sampling. Soil samples were stored in plastic bags at 4 °C before being passed through a 2-mm-mesh-width sieve for further analyses. Part of the soil and plant samples were dried in a 60 °C oven until reaching constant weight for water content determination. The water content of plants and soil was calculated using the following equation:

water content (g H_2_O g^−1^ DW) = (FW − DW)/DW,

where FW and DW represent the fresh weight and dry weight of plant (hydration) or soil (water content), respectively.

Tree height and basal diameter were determined in June 2024. For this purpose, 68 individual trees of *C. chengiana* and *P. orientalis* (34 of each) were selected at CJS, while 38 *P. orientalis* and 31 *C. chengiana* individual trees were selected at LHK. Trees were selected at the same study sites where leaf and soil samples were taken. The basal diameter was determined at 5 cm above the ground using a vernier caliper instead of the diameter at breast height, where the presence of numerous branches made direct measurement difficult. Tree height was measured using a tower ruler.

### 2.3. Biochemical Analyses

#### 2.3.1. Biochemical Analyses of Soil Samples

Soil organic matter was quantified using the potassium dichromate volumetric method with external heating [22]. Total nitrogen (N) and organic carbon (C) concentrations were determined by the macro-Kjeldahl method with a Kjeldahl nitrogen analyzer [23] and the dichromate oxidation–sulfate–ferrous titration method [24], respectively. Soil total phosphorus (P) was measured using the acid dissolution-molybdenum antimony colorimetric method after digestion in a mixture of sulfuric acid and perchloric acid [23]. The contents of C, N, and P are calculated and presented on a dry-weight basis.

#### 2.3.2. Biochemical Analyses of Leaf Samples

Hydrogen peroxide (H_2_O_2_) of leaf samples was extracted in 0.1% (*w*/*v*) trichloroacetic acid, and the absorbance was determined at 390 nm after reaction with 1 mol KI [25]. MDA was extracted in trichloroacetic acid, and the MDA content in the aqueous phase was calculated according to the absorbance recorded at 532 and 450 nm [26]. Total P contents were determined using the same method for soil P. Total C, N contents, as well as C and N isotopes, were measured with EA-IRMS (PYROCUBE coupled to an ISOPRIME IRMS, Elementar, Hanau, Germany) as previously described [27]. Soluble protein was extracted in Tris-HCl buffer and quantified using the colorimetric Bradford method as previously described [26]. Bovine serum albumin was used as a reference for quantification.

Low molecular weight metabolites were extracted and measured using an Agilent GC/MSD system consisting of an Agilent GC 7890A gas chromatograph (Agilent Technologies, Palo Alto, CA, USA) connected to a 5975C Inert XL EI/CI MSD quadrupole MS detector (Agilent Technologies) as described in Du et al. [25]. Peak detection and alignment were performed with the Quantitative Analysis Module of the Masshunter software (Agilent Technologies, Version B.07.00). Metabolites were normalized using the peak area of the internal standard, ribitol, and the dry weight of samples and presented as relative abundances. Signals corresponding to artefacts were omitted according to the analysis of ‘blank’ samples prepared in the same manner as biological samples. Total soluble sugar and amino acid contents in the extract for metabolome analysis were calorimetrically determined using the anthrone–sulfuric acid method and ninhydrin method, respectively [25,28].

### 2.4. Statistical Analysis

Statistical analyses were performed using SigmaPlot 12.0 (Systat Software GmbH, Erkrath, Germany). For leaf samples, differences between the two species within the same site and differences between the two sites of the same species were determined by Student’s t-test. For soil samples, the same method was performed to examine differences between the two layers (0–20 and 20–40 cm) within the same site and differences between the two sites of the same layer. Data were transformed by denary logarithm to match normal distribution when necessary. Data shown in the figures represent means ± SD of 6 plants (n = 6) on a dry weight basis. To have an overview of the species-specific and site-related effects, PLS-DA (partial least square discriminant analysis) was conducted using a public web tool (MetaboAnalyst 6.0, https://www.metaboanalyst.ca/MetaboAnalyst/ (accessed on 17 July 2024) [29] after log10 transformation and mean-centering. Missing values were replaced by 1/5 of the minimum abundance of respective compounds, assuming that their concentrations were below the detection limit.

## 3. Results

### 3.1. Soil Properties at the Two Field Sites

**Figure 1:**
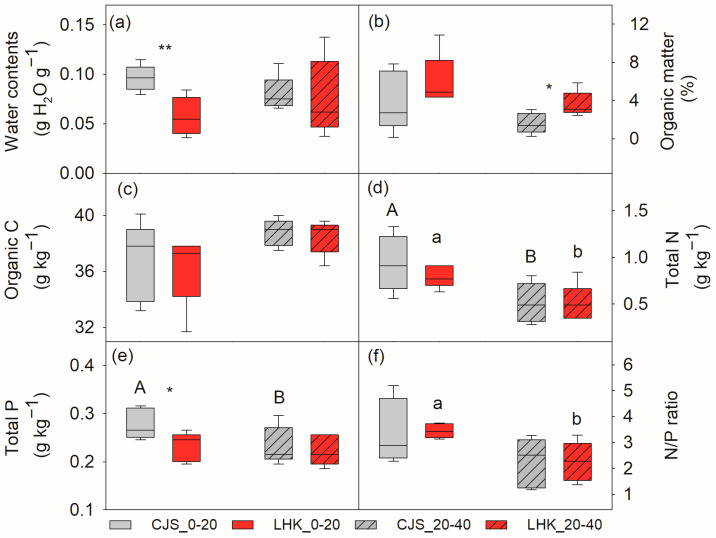
Soil water content (**a**), organic matter (**b**), organic carbon (**c**), total nitrogen (**d**), total phosphorus (**e**), and nitrogen to phosphorus (N/P) ratio (**f**) at the field sites of Cuojishan (CJS) and Lianghekou (LHK). Soil parameters were determined at two depths of 0–20 cm (without hatching) and 20–40 cm (hatched bar). Asterisks indicate significant differences between the two sites within the same depth (*, *p* < 0.05; **, *p* < 0.01). Different upper-case and lower-case letters indicate significant differences between the two soil depths at CJS and LHK, respectively. Data shown means ± SE (n = 6) on a dry weight basis.

Water and total P was significantly lower in the top layer soil (0–20 cm) at LHK compared to CJS, i.e., 5.7 ± 1.0 compared to 9.6 ± 0.6 g H_2_O g^−1^ soil (*p* = 0.009), and 0.23 ± 0.01 compared to 0.28 ± 0.01 g kg^−1^ soil (*p* = 0.022), respectively (Figure 1a,e). Soil organic matter, organic C, and total N contents were similar at both sites (Figure 1b–d). In the deeper layer soil (20–40 cm), no significant difference in water, organic C, and total N and P contents were found between the two filed sites, except for organic matter, which was significantly higher at LHK (3.6 ± 0.5%) compared to CJS (1.6 ± 0.4%) (*p* = 0.014) (Figure 1b).

### 3.2. Tree Growth

**Figure 2:**
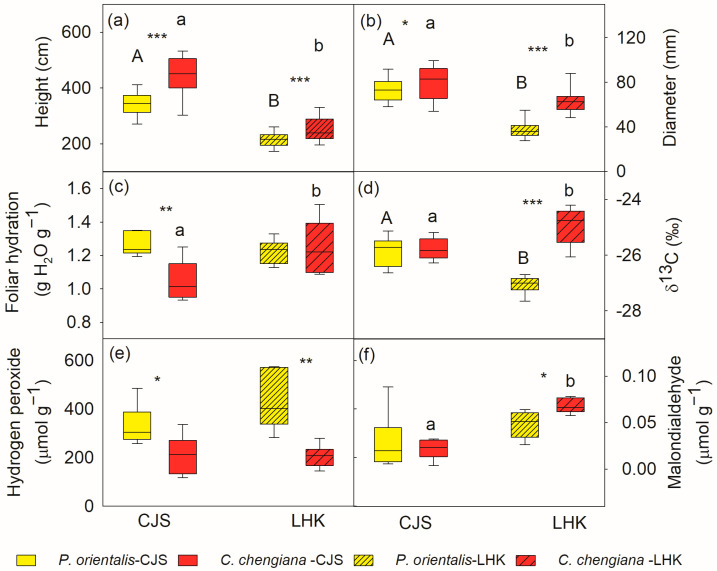
Tree height (**a**), diameter at 5 cm above ground (**b**), leaf hydration (**c**), δ13C (**d**), hydrogen peroxide (**e**) and malondialdehyde contents (**f**) of *Platycladus orientalis* (yellow) and *Cupressus chengiana* (red) at Cuojishan (CJS, right panel, without hatching) and Lianghekou (LHK, left panel, hatched bars). Asterisks indicate significant differences between the two species within the same site (*, *p* < 0.05; **, *p* < 0.01; ***, *p* < 0.001). Different upper-case and lower-case letters indicate significant differences between the two sites within the same species, respectively. Data shown means ± SE (n = 6 for **c**–**f**, n = 31–38 for **a**,**b**) on a dry weight basis.

The results indicated that *C. chengiana* trees grew faster than *P. orientalis* trees (Figure 2). *C. chengiana* trees were 30% and 20% higher (*p* < 0.001) than *P. orientalis* trees at CJS and LHK, respectively (Figure 2a). The diameter at 5 cm above ground of *C. chengiana* trees was also significantly higher than that of *P. orientalis* trees, i.e., 8% (*p* = 0.028) at CJS and 67% higher (*p* < 0.001) at LHK (Figure 2b).

### 3.3. Species-Specific Differences at Two Sites

In addition to different growth rates, differences between the two species were also demonstrated in plant water relations, reactive oxygen species (ROS) levels, and metabolome profiles. Foliar hydration of *P. orientalis* trees was 21% higher (*p* = 0.002) than *C. chengiana* trees at CJS, while this was similar at LHK (Figure 2c). Foliar δ13C signatures in both species were similar at CJS, but they were significantly enriched in *C. chengiana* trees and depleted in *P. orientalis* trees at LHK (Figure 2d). Leaf H_2_O_2_ contents of *P. orientalis* trees were significantly higher than *C. chengiana* trees at both sites, i.e., 57% and 91% higher at CJS and LHK, respectively (Figure 2e). MDA contents were 20% higher in *C. chengiana* trees than in *P*. *orientalis* trees at LHK (Figure 2f).

**Figure 3:**
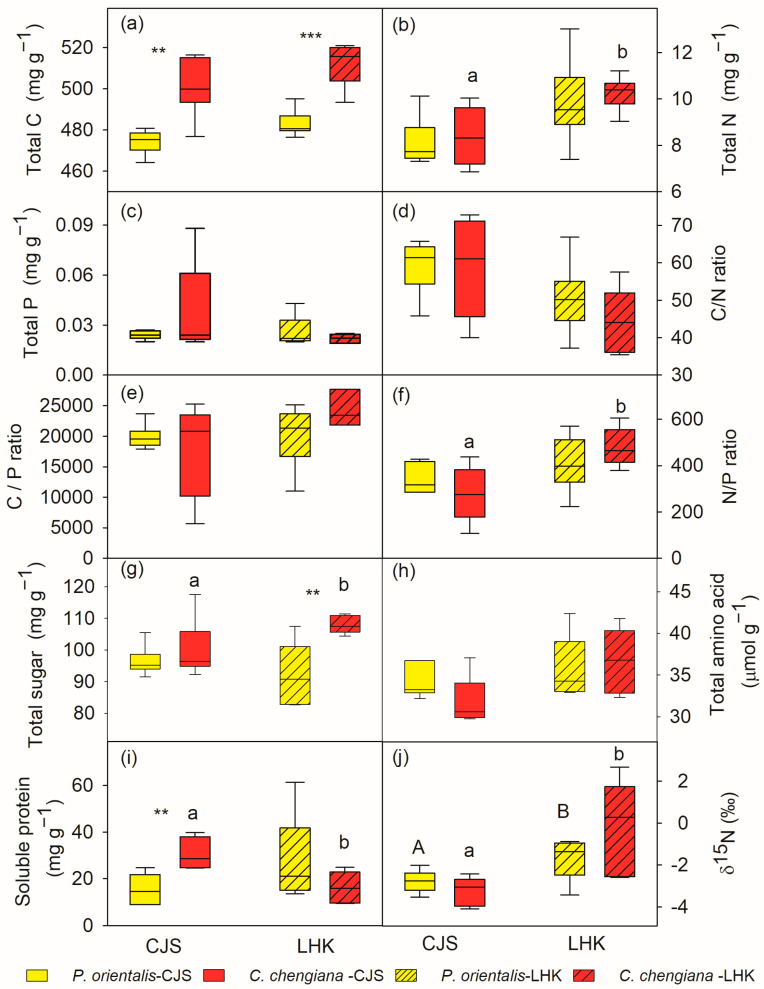
Total carbon (C), nitrogen (N), phosphorus (P) contents and their ratios (**a**–**f**), soluble sugar (**g**), amino acid (**h**), soluble protein (**i**), and δ15N (**j**) in leaves of *Platycladus orientalis* (yellow) and *Cupressus chengiana* (red) at Cuojishan (CJS, right panel, without hatching) and Lianghekou (LHK, left panel, hatched bars). Asterisks indicate significant differences between the two species within the same site (**, *p* < 0.01; ***, *p* < 0.001). Different upper-case and lower-case letters indicate significant differences between the two sites within the same species, respectively. Data shown means ± SE (n = 6) on a dry weight basis.

Total C contents in *C. chengiana* leaves were significantly (*p* < 0.001) lower than *P. orientalis* leaves at both sites (9% and 8% at CJS and LHK, respectively) (Figure 3a). No significant differences in leaf total N, P, as well as C/N, C/P, and N/P ratios, total amino acids, and δ^15^N values, were found between the two species at both sites (Figure 3b–f,h,j). While the foliar total sugar content of *C. chengiana* trees was 28% higher than that of *P. orientalis* trees at LHK (*p* = 0.002), it was similar at CJS (Figure 3g). Soluble protein content in *C. chengiana* leaves was 256% higher than in *P. orientalis* leaves at CJS (*p* = 0.001) (Figure 3i).

**Figure 4:**
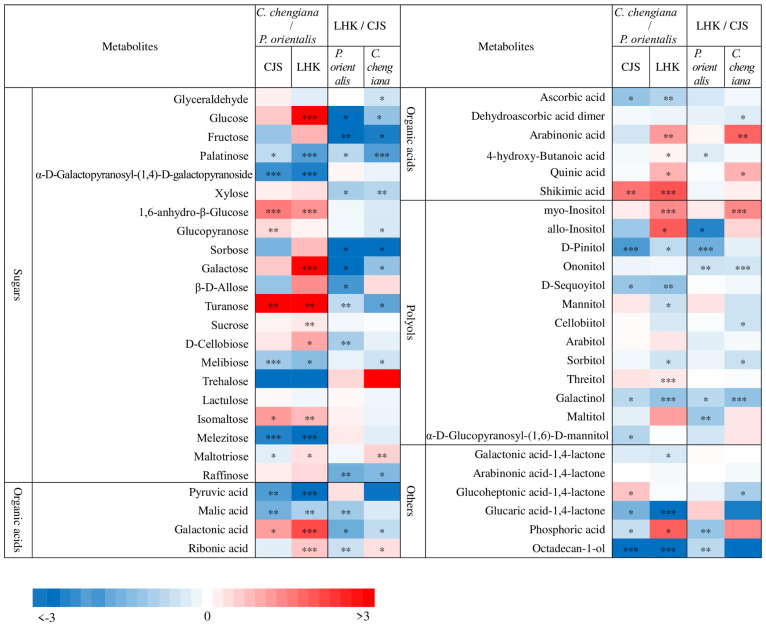
Fold change (log2) of sugars, organic acids, polyols, and other compounds in leaves of *Platycladus orientalis* and *Cupressus chengiana* between Lianghekou (LHK) and Cuojishan (CJS) (**left panels**) and between *C. chengiana* and *P. orientalis* at CJS and LHK (**right panels**). Asterisks indicate significant differences between CJS and LHK within the same species and between the two species within the same site (*, *p* < 0.05; **, *p* < 0.01; ***, *p* < 0.001). Data shown means ± SE (n = 6) on a dry weight basis.

**Figure 5:**
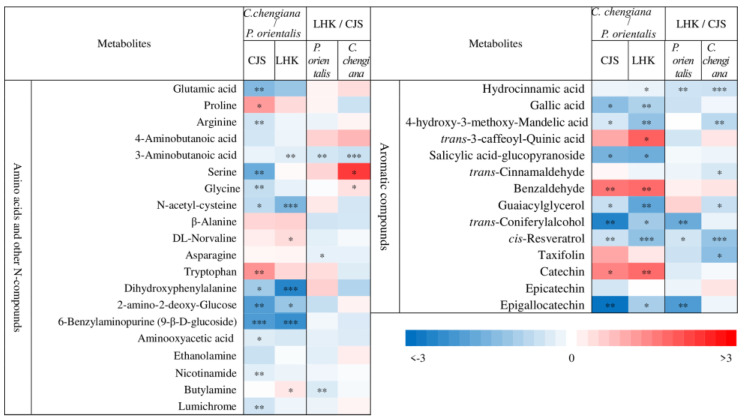
Fold change (log2) of amino acids and other N-compounds and aromatic compounds in leaves of *Platycladus orientalis* and *Cupressus chengiana* between Lianghekou (LHK) and Cuojishan (CJS) (**left panels**) and between *C. chengiana* and *P. orientalis* at CJS and LHK (**right panels**). Asterisks indicate significant differences between CJS and LHK within the same species and between the two species within the same site (*, *p* < 0.05; **, *p* < 0.01; ***, *p* < 0.001). Data shown means ± SE (n = 6) on a dry weight basis.

Abundances of low molecular weight compounds differed between the two species (Figure 4 and Figure 5). Compared to *P. orientalis*, *C. chengiana* had significantly lower abundances of palatinose, α-D-galactopyranosyl-(1,4)-D-galactopyranoside, melibiose, trehalose, and melezitose, but higher 1,6-anhydro-β-glucose, turanose and isomaltose abundances at both sites (Figure 4). No significant differences in glucose, galactose, sucrose, and D-cellobiose abundances were observed at CJS, but they were significantly higher in *C. chengiana* leaves at LHK. Pyruvic acid, malic acid, and ascorbic acid were less abundant, while galactonic acid and shikimic acid were more abundant in *C. chengiana* than in *P. orientalis* at both sites. Abundances of ribonic acid, arabinonic acid, 4-hydroxy-butanoic acid, and quinic acid were significantly higher in *C. chengiana* than in *P. orientalis* at LHK. In contrast to mannitol and sorbitol, the abundances of myo-inositol, allo-inositol, and threitol were higher in *C. chengiana* leaves but only at LHK. D-Pinitol, D-sequoyitol, galactinol, glucaric acid-1,4-lactone, and octadecan-1-ol were less abundant in *C. chengiana* than in *P. orientalis* at both sites. Phosphoric acid was more abundant in *C. chengiana* leaves at CJS but less abundant at LHK (Figure 4). Generally, amino acids and several other N compounds were less abundant in *C. chengiana* leaves than in *P. orientalis* leaves, except for proline, tryptophan, β-alanine, and DL-norvaline, which showed generally higher abundances in *C. chengiana* leaves (Figure 5). In contrast to trans-3-caffeoyl-quinic acid, benzaldehyde, taxifolin, and catechin, the abundance of other aromatic compounds was also lower in *C. chengiana* leaves at both sites (Figure 5).

### 3.4. Differences between the Two Sites

**Figure 6:**
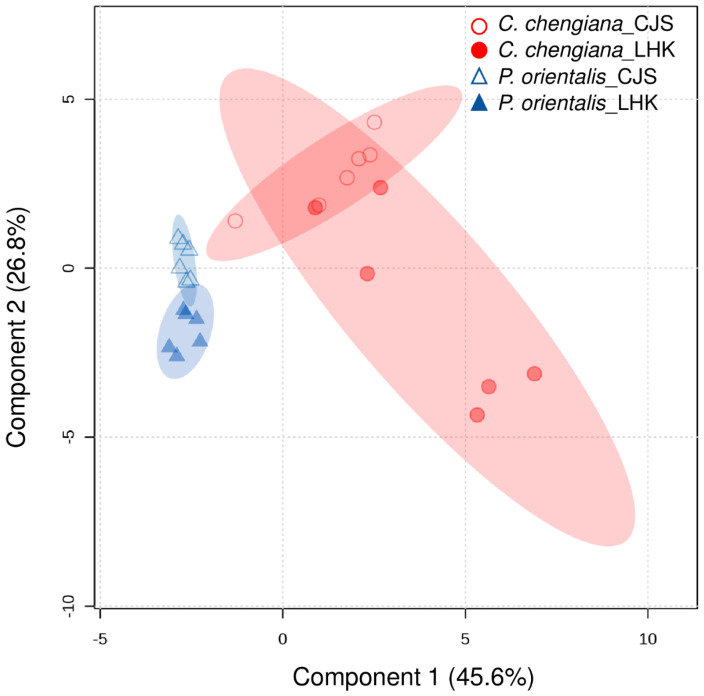
Clustering of all physiological and metabolic parameters in leaves of *Platycladus orientalis* (triangle) and *Cupressus chengiana* (circle) at Cuojishan (CJS) and Lianghekou (LHK). Semi-transparent shadings indicate 95% confidence regions.

**Figure 7:**
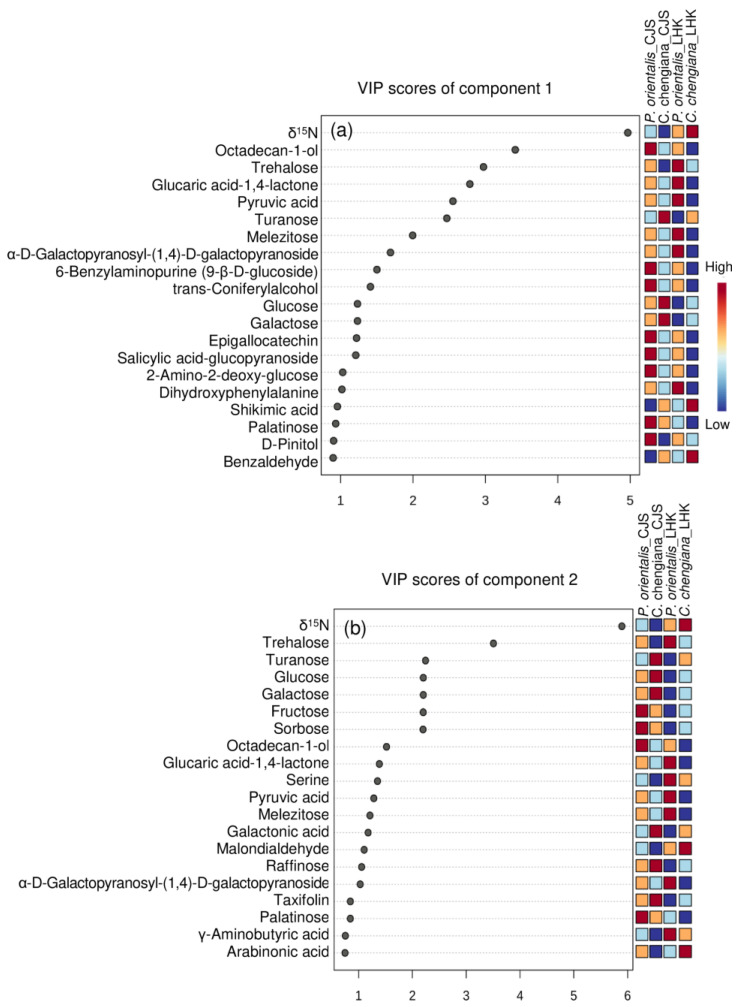
Top 20 parameters of component 1 (upper panel, **a**) and component 2 (lower panel, **b**) according to the VIP scores of PLS-DA analysis.

The site also had notable effects on the foliar physiological traits of both species (Figure 2, Figure 3, Figure 4 and Figure 5). In *P. orientalis* trees, except δ15N, concentrations of most of the other compounds were lower at LHK in comparison to trees at CJS (Figure 2, Figure 3, Figure 4 and Figure 5). In contrast, in *C. chengiana* trees, foliar hydration, contents of MDA, total N, total sugar, trehalose, maltotriose, serine, glycine, arabinonic acid, quinic acid, myo-inositol, as well as δ13C, δ15N signatures and N/P ratio were higher at LHK than trees at CJS. Whereas contents of soluble protein, most sugars, polyols, and aromatic compounds were less abundant in leaves of *C. chengiana* trees at LHK. Less site effects on amino acid abundances, except for higher serine and glycine and lower 3-aminobutanoic acid concentrations, were observed at LHK in comparison to CJS (Figure 2, Figure 3, Figure 4 and Figure 5). PLS-DA analysis based on all the biochemical parameters of the two species showed apparent species- and site-determined clusters (R^2^ = 0.93, Q^2^ = 0.63) (Figure 6). The species-specific effect was separated along component 1, while the site-specific effect was mainly separated along component 2. The two components explained 72.4% of the total variance, and nitrogen and sugars largely determined the two components (Figure 7).

## 4. Discussion

The growth potential of trees and their related physiological mechanisms are of particular importance for afforestation in drylands. In the present study, we found there were complex physiological and metabolic regulations behind the higher growth rate of *C. chengiana* trees in comparison to *P. orientalis* trees in a typical dryland in the hot and dry Minjiang Valley, China.

### 4.1. Different Growth Rates of the Two Species

Both *C. chengiana* and *P. orientalis* are widely used tree species for afforestation in Minjiang Valley [17,30,31]. Previous studies have shown both species were highly resistant to adverse environmental conditions, such as poor soil nutrients, limited water availability, and temperature extremes [18,32]; however, knowledge of species growth and metabolomics is scarce. In line with other studies, soil water and nutrient contents were much lower at LHK than at CJS (Figure 1), especially the top soil layer [13,14]. Our results show that *C. chengiana* trees grew faster than *P. orientalis* trees at both sites (Figure 2). In accordance with the higher growth of *C. chengiana* trees, total C and soluble sugar contents in *C. chengiana* leaves were higher than in *P. orientalis* trees, particularly at the hotter and dryer site of LHK (Figure 3), which may indicate a greater assimilation rate and/or lower resources consumption due to well-tuned resistance to dry and hot conditions in the hostile habitat [33,34]. This is further supported by the significantly lower H_2_O_2_ contents at both sites (Figure 2e), which may also support the higher growth rate due to less energy and assimilated input for ROS formation and scavenging [35]. However, little is known about the enzymatic and non-enzymatic ROS scavenge systems, as well as the difference between the two species [36,37,38], and therefore, they deserve further studies. It is apparent that the protection from increased antioxidants of polyols (i.e., myo- and allo-inositol, threitol), amino acids (i.e., proline and tryptophan), and aromatics (e.g., catechin and benzaldehyde) cannot be excluded [39,40,41,42,43]. The ascorbic acid concentration was lower in *C. chengiana* leaves at both sites, probably at least partly due to limited biosynthesis from the D-mannose/L-galactose pathway as seen from the lower abundance of its precursor of galactonic acid-1,4-lactone (Figure 4) [44]. Although we did not determine the contents of the antioxidant glutathione [45], a lower glutathione content in *C. chengiana* tree leaves was also proposed from the low abundance of its precursor N-acetyl-cysteine (Figure 5). Compared to *P. orientalis* trees, lower foliar hydration observed in older *C. chengiana* trees at CJS was not found in younger trees at the drier and hotter site where the δ^13^C signature was less negative (*p* < 0.001) (Figure 2c,d). Similarly, less negative foliar δ^13^C signatures were also documented in other evergreen species under drought conditions, for instance, Douglas fir (*Pseudotsuga menziesii*) [46] and silver fir (*Abies alba* Mill.) [47]. It seems that *C. chengiana* trees tended to increase stomatal closure to prevent water loss [48] as observed by other studies [36,38], which was not observed in *P. orientalis* trees as indicated by the more negative foliar δ^13^C values at LHK compared to CJS.

### 4.2. Climate Conditions Mediated Species-Specific Differences of Metabolome

Foliar metabolome profiles depend highly on species and are strongly affected by various biotic and abiotic conditions [49,50]. The apparent site-related species-specific ROS homeostasis [37], as well as C and N fractions, are reflected in individual metabolites, e.g., soluble sugars, organic acids, amino acids, and other N compounds (Figure 4, Figure 5 and Figure 7). Soluble sugars, especially glucose, fructose, and sucrose, act as nutrients and signaling molecules. They play an apparent central role in plant structure and metabolism and are involved in the responses to various stresses [51]. In the present study, apart from δ15N, sugars contributed significantly to the clustering of the two species, as shown in the VIP scores plot of the top 20 parameters (Figure 7). In general, *C. chengiana* trees had higher soluble sugar contents, particularly at the drier and hotter site of LHK (*p* < 0.01). The strong increase in soluble sugar was mainly attributed to higher abundances of glucose, galactose, sucrose, D-cellobiose, fructose, isomaltose, and raffinose (Figure 4). Together with the declined abundances of intermediates sorbitol and melibiose, the metabolisms of site-mediated species-specific starch, sucrose, and galactose were projected. In addition, *C. chengiana* trees accumulated 1,6-anhydro-beta-glucose and turanose, whereas *P. orientalis* trees had higher foliar levels of trehalose as well as palatinose, melibiose, and melezitose irrespective of age and environmental conditions (Figure 4). The disaccharide trehalose mediates several biochemical, physiological, and molecular processes in plants and is involved in plant tolerance against different stresses, including drought and heat [52]. *C. chengiana* trees may actively control trehalose at a low level to prevent the inhibitory effects of high trehalose on growth, as observed in other plant species [53].

The two most abundant essential elements in plants are C and N, and their metabolisms are tightly coupled through the tricarboxylic acid (TCA) cycle and are closely involved in biotic and abiotic stress responses and cellular redox homeostasis [54]. The TCA cycle intermediates provide essential precursors for respiration and N metabolism, and their accumulation is extremely variable between species [55]. In the present study, we found pyruvic acid and malic acid were significantly lower in *C. chengiana* leaves than in *P. orientalis* leaves at both sites. In line with the declined abundances of organic acids involved in the TCA cycle, abundances of most amino acids and other N-compounds also tended to decrease except DL-norvaline and butylamine at LHK and proline and tryptophan at CJS, indicating species-specific N metabolism. In general, abundances of the glutamate family amino acids, i.e., glutamic acid, arginine, 4-aminobutanic acid (GABA), and its isomer of 3-aminobutanic acid [56] were lower in *C. chengiana* leaves than in *P. orientalis* leaves. Similar patterns were also observed in the amino acids involved in photorespiration, i.e., glycine, serine, and the precursor N-acetyl-cysteine to cysteine, indicating higher photorespiration in *P. orientalis* leaves [57]. It is worth pointing out that since we took the samples in autumn, the differences in C and N metabolites between the two species may also be attributed to translocation from leaves to other parts of the trees [58,59], which was not considered in the present study.

Secondary metabolites, as natural byproducts of primary metabolic processes, play a significant role in plant defense and, therefore, normally accumulate under stressed conditions. And its production and accumulation vary from species to species and are mediated by environmental conditions [60]. In the present study, higher foliar abundances of quinic acid, trans-3-coffeoyl-quinic acid, shikimic acid, as well as the aromatic amino acid tryptophan, the precursors of a variety of secondary metabolites observed in *C. chengiana* trees were not translated into higher abundances of most identified aromatic compounds. This may contribute to the higher input of C from these secondary metabolites synthesis into metabolites supporting the growth and biomass production of *C. chengiana* trees (Figure 2 and Figure 3) [61]. For example, biomass growth was negatively correlated with lignin content in both angiosperms and gymnosperms tree species, and sucrose may be a key regulator of growth and lignin production, especially the competition for C allocation between lignin and cellulosics [62]. Moreover, we also found benzaldehyde was significantly higher in *C. chengiana* leaves than in *P. orientalis* leaves. Studies have shown that this simplest aromatic aldehyde plays important roles in chemical communications as well as biotic and abiotic stress defense [41,63]. Further studies aiming to reveal the complex secondary metabolism of the two species are required.

Plants are subjected to a variety of environmental stresses, including elevated temperatures and limited water availability, which is particularly prominent in hot and dry valleys [7]. In the present study, both tree species reacted similarly in response to the hotter and drier conditions, i.e., declined abundances of most soluble sugars, organic acids, polyols, and aromatic compounds at LHK in comparison to CJS. Generally, stronger effects of hotter and drier conditions on *C. chengiana* trees were expected, as seen from the large differences between the two field sites. For example, higher concentrations of the osmoprotectant trehalose [52,53], maltotriose—indicating starch breakdown [64], serine and glycine—involved in photorespiration [57], myo-inositol—crucial for development and signaling in plants [65,66]—observed at LHK might collectively contribute to better coping with the hassle conditions [7,67] in addition to stomatal control. We also found that leaf δ^15^N values of the two species were both higher at LHK than CJS (Figure 3j), which reflected the difference of δ^15^N in soil N source [68]. Stimulated losses of N through ammonia, nitrate leaching, or denitrification at the LHK site are speculated [69]. A negative correlation between foliar δ^15^N and mean annual precipitation was also found across a wide range of ecosystems [70]. This may be attributed to the adaptation of preference N sources of the trees, i.e., the trees took up more δ^15^N enriched ammonium than δ^15^N depleted nitrate at the drier site LHK, and higher fungal discrimination at the CJS site [13,14,69]. It appears that the trees at the drier and hotter site LHK have been efficient in their nitrogen uptake from soil despite the low soil N concentration [14]. This observation was in line with the slightly higher total N and total amino acids contents (Figure 3), which indicates that the trees at LHK gained more nitrogen either for maintaining an efficient photosynthetic capacity within the leaves and/or for coping with the stressful conditions [71,72,73]. One possible mechanism for the observed higher nitrogen uptake is the warmer climate-enhanced activity of ectomycorrhizal fungi, which can obtain nitrogen from organic sources and deliver it to the host plant [74,75,76]. Yang et al. [77] showed that aboveground plant biomass could be a key factor driving the changes in soil organic C, total N, and arbuscular mycorrhizal fungal abundance in arid areas in China. Therefore, the present results also highlight the requirement of N input management practice in a long-term view of afforestation in similar arid areas [78]. We have to be aware that the differences within each species between the two field sites can also be due to both climate conditions and age effects since the trees at LHK are younger and smaller than those at CJS [79,80,81].

## 5. Conclusions

In summary, the results from this study suggest that *C. chengiana* grows faster than growth *P. orientalis* irrespective of site conditions at the Minjiang hot and dry valley in Southwest China. Trees of *C. chengiana* tended to close stomata under dry and hot conditions to prevent water loss. Differences between the two species were also observed in ROS levels, carbon and nitrogen fractions, and secondary metabolites, and they were mediated by climate conditions at the two sites. Our results, for the first time, compare the performance and metabolic profiles of two important afforestation cypress species, highlighting the species-specific metabolic regulations in response to stressful conditions. Considering that both trees are long-lived species, the growth advantage of *C. chengiana* over *P. orientalis* is likely to increase further with age. However, more research should be done, for example, on suitable areas and local adaptations before large-scale afforestation with *C. chengiana*.

## Data Availability

The data presented in this study are available on request from the corresponding author.
